# Supplementary material for: Protein Disulfide Isomerase FgEps1 Is a Secreted Virulence Factor in Fusarium graminearum
Source: J Fungi (Basel). 2023 Oct 12;9(10):1009. doi: 10.3390/jof9101009 (PMC10607971; doi:10.3390/jof9101009)
Supplement: Supplementary file 1 [file jof-09-01009-s001.zip › Supplementary Material Note.pdf]

Supplementary Materials: Table S1: PCR primers used in this study; Figure S1: A schematic diagram of homologous recombination to construct a deletion mutant of *FgEps1*. Figure S2: Validation of the ΔFgEps1 deletion mutant by PCR. （A） A schematic diagram of ΔFgEps1 deletion mutant primer validation is presented.（B）Lanes 1-4 show the identification of ID-F/HYC-R, with lane 4 being the wild type; lanes 5-8 show the identification of ID-R/HYC-F, with lane 8 being the wild type; lanes 9-12 show the identification of ID-F/ID-R, with lane 12 being the wild type; lanes 13-16 show the identification of HYC-F/HYC-R, with lane 16 being the wild type.（C）The relative gene expression of wild type and three. Figure S3: Statistics on the number of septa in the conidia produced by each strain. Figure S4: Phylogenetic tree of Eps1 proteins in different fungi. Figure S5: Quantification of cell death by measuring electrolyte leakage 3 and 5 dpa. Means and SEs were calculated from three independent experiments. The statistical analyses were performed with Student's t-test. Bars indicate ±SE. *p <0.05, ***p <0.001; Figure S6: The colony morphology of three *ΔFgEps1* mutant transformants under different kinds of pressure. *ΔFgEps1* transformants at the transcriptional level.
