# Supplementary material for: Protein Disulfide Isomerase FgEps1 Is a Secreted Virulence Factor in Fusarium graminearum
Source: J Fungi (Basel). 2023 Oct 12;9(10):1009. doi: 10.3390/jof9101009 (PMC10607971; doi:10.3390/jof9101009)
Supplement: Supplementary file 1 [file jof-09-01009-s001.zip › Table S1.pdf]

Usage	Name	Primer Sequences(5'-3')
Gene knockout and identification of FgEps1	UP-F	TATGCGGTTCTTATCTTCATG
	UP-R	CAAAATAGGCATTGATGTGTTGACCTCCCAACTTCGAGACCACGTTCTGTAT
	DOWN-F	CTCGTCCGAGGGCAAAGGAATAGAGTAGTTGTGTCATCTGCGTAATGG
	DOWN-R	AGCAGCAGTCTTACCAGCAGTA
	ID-F	CGAATGCGCATCAATGAGGA
	ID-R:	TCTCACTGATGTGCTCTACAC
Construction of signal peptide secretion vector	Psuc2-Eps1-F	TCCAAGCTCGGAATTTTAATTAAGAATTCATGCGTTTCTCTCCGCTTGC
	Psuc2-Eps1-R	CGACTCACTATAGGGAGAACCTCGAGGTCAGCCAGAACAAACGCTG
Construction of plant transient expression vector	pBin-Eps1-F	CCCCGGGGTCGACGGATCCATGCGTTTCTCTCCGCTTGC
	pBin-Eps1-R	CTCTAGTTCATCTAGAGGATCCATCAGACTTGGCGTTGCCAT
The expression of FgEps1 during infection	Ex-Eps1-F	AATATGACGGTATTGACGAC
	Ex-Eps1-R	TAACATCATCGAGAGATTTG
Hygromycin fragment amplification	HYC-F	GGAGGTCAACACATCAATGCCTATT
	HYC-R	CTACTCTATTCCTTTGCCCT
Construction of complementary carrier	np-Eps1-GFP-F	ACTCACTATAGGGCGAATTGGGTACTCAAATTGGTTACGTCTCTGGTGAGAGGAGCG
	np-Eps1-GFP-R	CACCACCCCGGTGAACAGCTCCTCGCCCTTGCTCACTAGGTTGCCGCCCTTTTCC
Expression of Tri gene cluster members	RT Tri1-F	GATGTTCCTTCTCGACAGCGT
	RT Tri1-R	CACTGGTCGAAGATAGCTGG
	RT Tri3-F	TGTTACGATCAATGGCTTGG
	RT Tri3-R	TCCTCGTTGTAGTTTGCATCA
	RT Tri4-F	ACGTGTGGCTACTCAGGAGA
	RT Tri4-R	TGGAATTGCCTTGGGGTA
	RT Tri5-F	ATGGCGGATCTATCTATTCAC
	RT Tri5-R	CCATTCATACGACGAAGGAAT
	RT Tri6-F	AAATGCCCATTCCCTAGTTG
	RT Tri6-R	ATCTCGCATGTTATCCACCCT
	RT Tri7-F	TACCGTCGTCTTCAAAACCA
	RT Tri7-R	ACGCCAATGGTGTTCACAAA
	RT Tri8-F	ATATAACGGTACCCCCAGATG
	RT Tri8-R	TGTTTGTAGGACACTTCCGGT
	RT Tri10-F	TCCCAACCTTTCAGAGGTTCA
	RT Tri10-R	TGATCCGTCAAGTCTTCCCAT
	RT Tri11-F	TGAGAACGACATGTGGGCAAT
	RT Tri11-R	AGGCTTGTTCCATGCAAGAT
	RT Tri12-F	ACGAACAGCACTGCTACGGT
	RT Tri12-R	TTCCTGCTTGTGACTCCAAT
	RT Tri13-F	ACGCAGATCCTGGGATATCA
	RT Tri13-R	CAGCCCAGTATTTTGCCAAA
	RT Tri14-F	AACTCCCGTTGTGATCAAGCA
	RT Tri14-R	AACAGTAATGTTGGCACCGT
	RT actin-F	ATCCACGTCACCACTTTCAA
	RT actin-R	TGCCTTGAGATCCACATTTG
