# Supplementary figures and images for: Protein Disulfide Isomerase FgEps1 Is a Secreted Virulence Factor in Fusarium graminearum
Source: J Fungi (Basel). 2023 Oct 12;9(10):1009. doi: 10.3390/jof9101009 (PMC10607971; doi:10.3390/jof9101009)

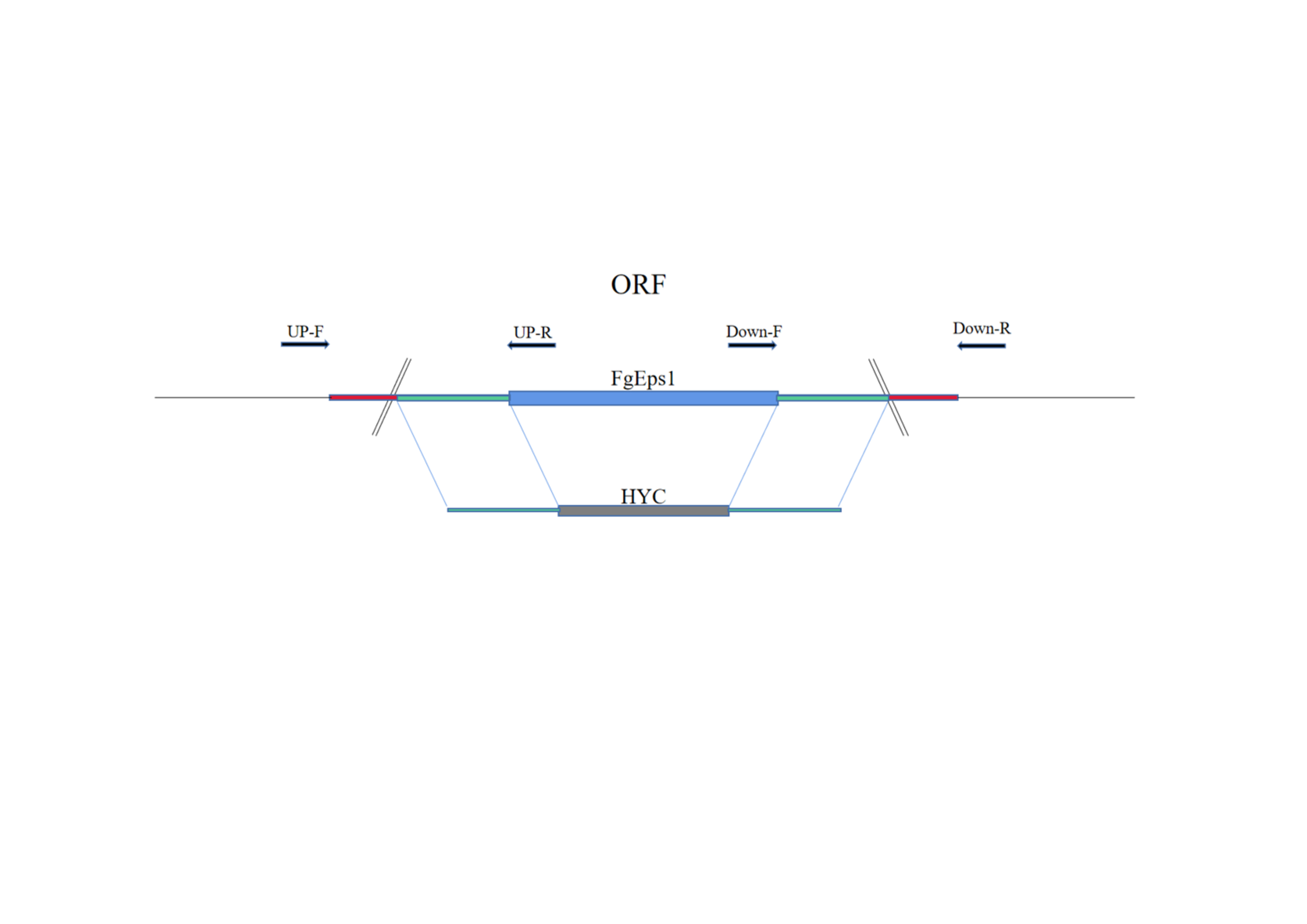

Supplement: Supplementary file 1 [file jof-09-01009-s001.zip › Figure S1.PNG]

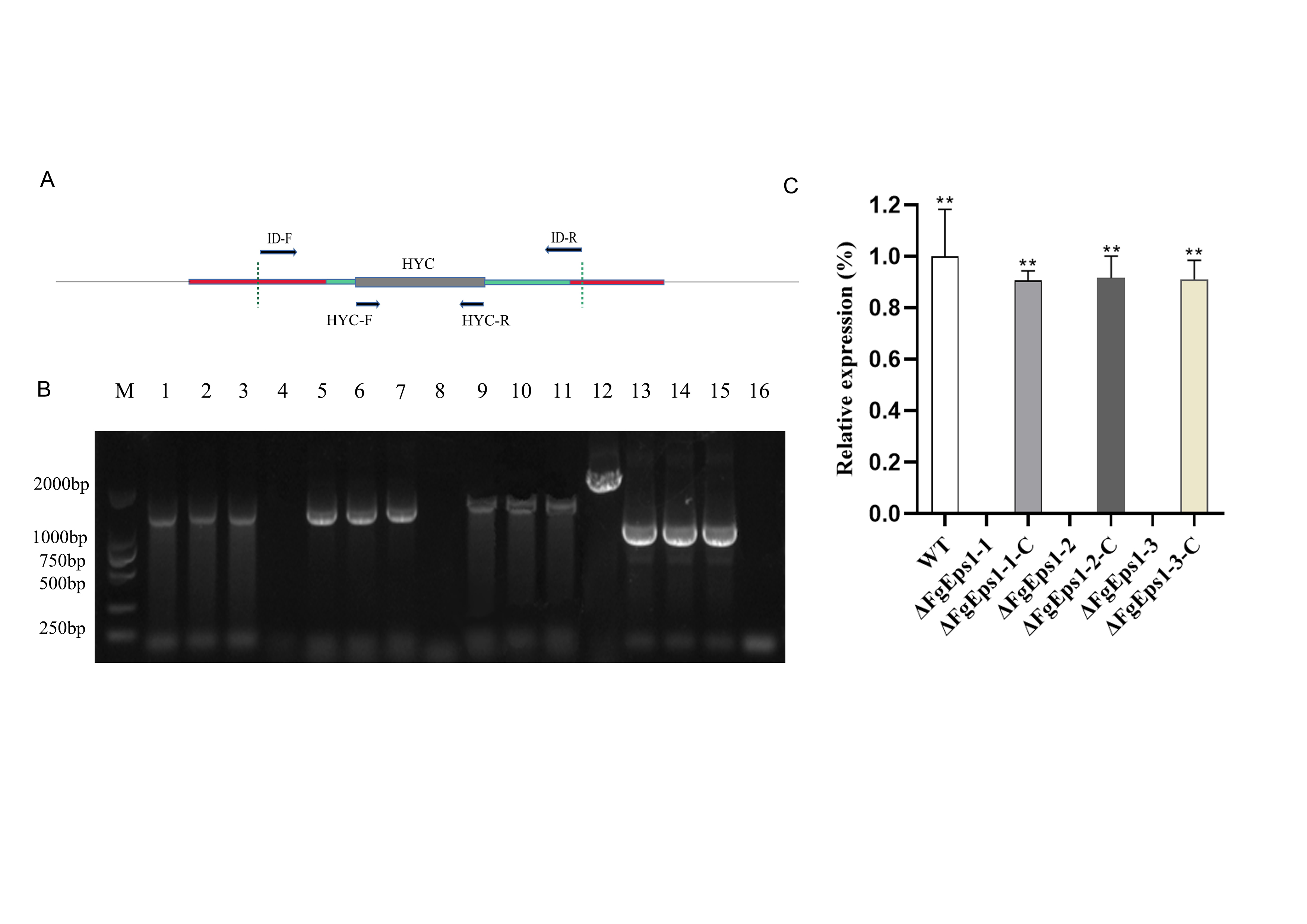

Supplement: Supplementary file 1 [file jof-09-01009-s001.zip › Figure S2.PNG]

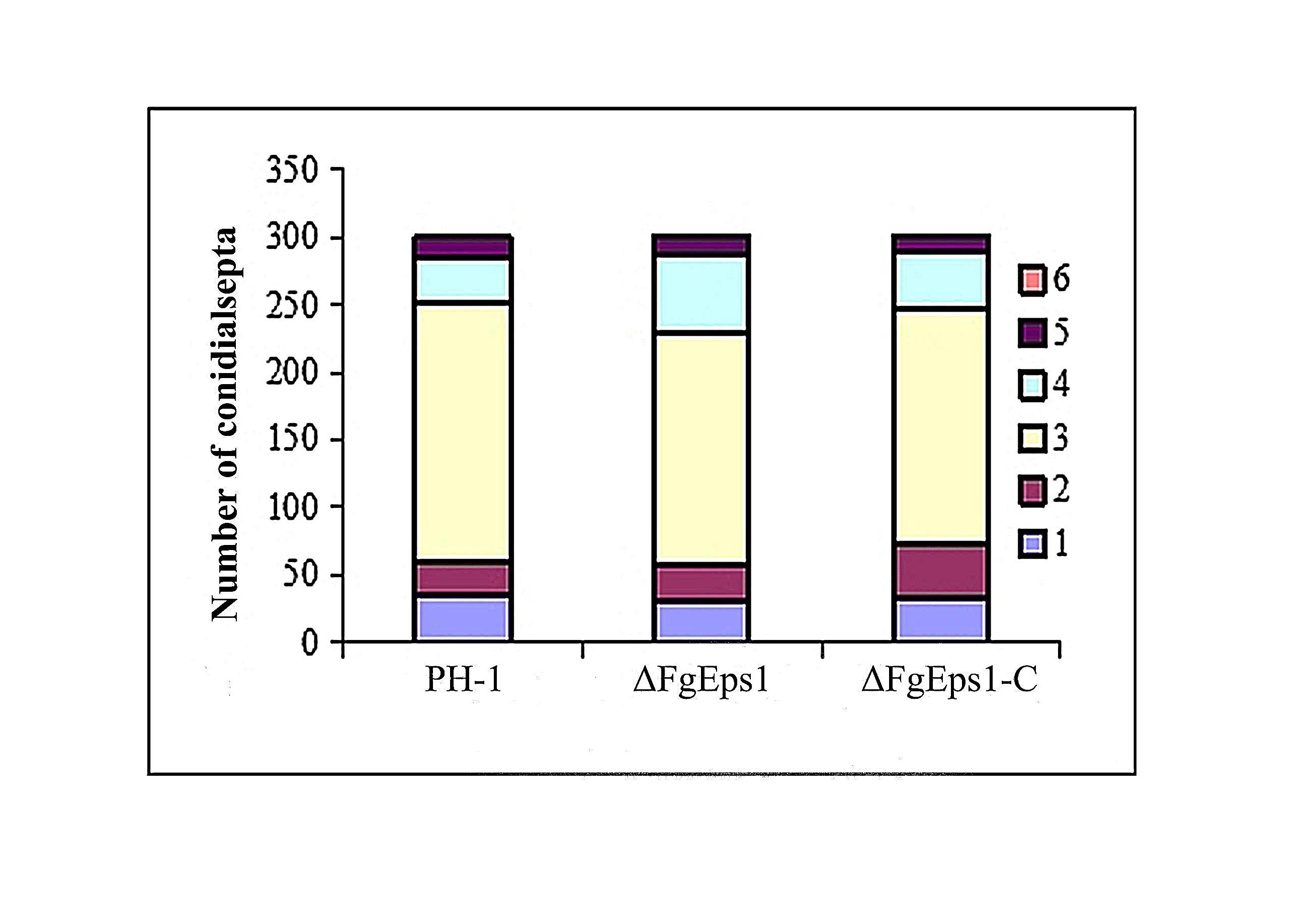

Supplement: Supplementary file 1 [file jof-09-01009-s001.zip › Figure S3 .PNG]

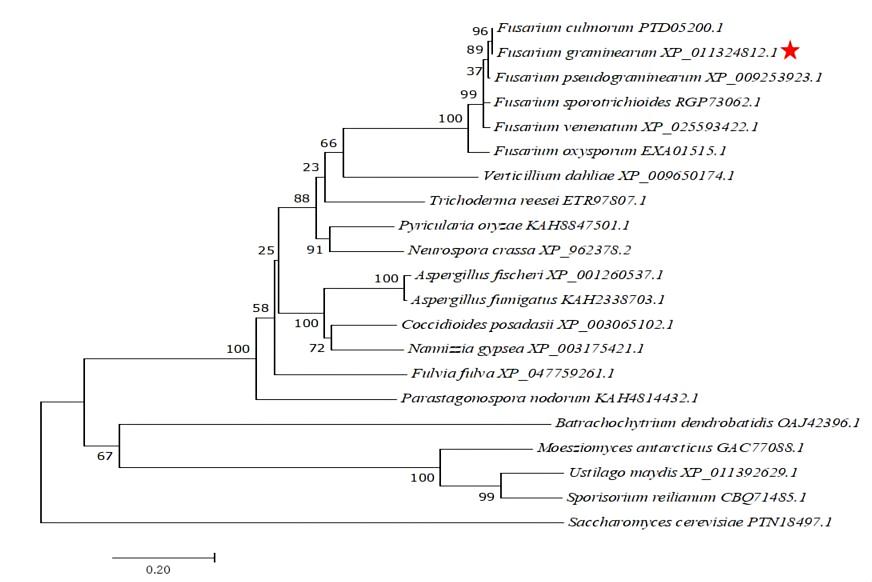

Supplement: Supplementary file 1 [file jof-09-01009-s001.zip › Figure S4.PNG]

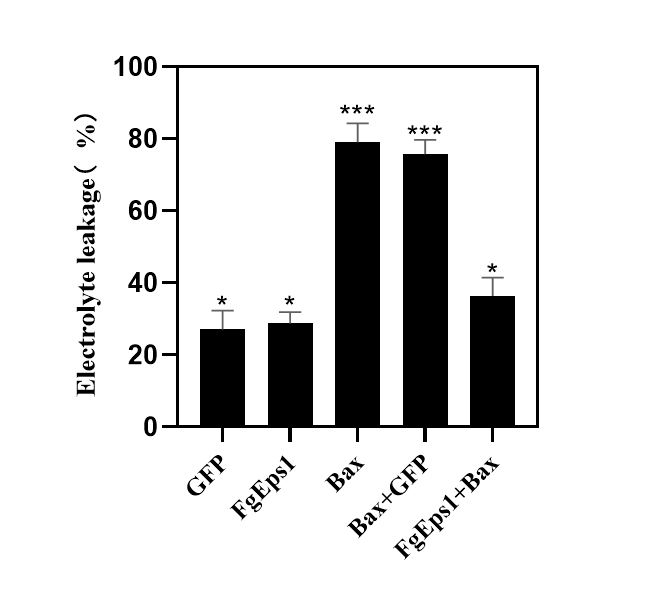

Supplement: Supplementary file 1 [file jof-09-01009-s001.zip › Figure S5.PNG]

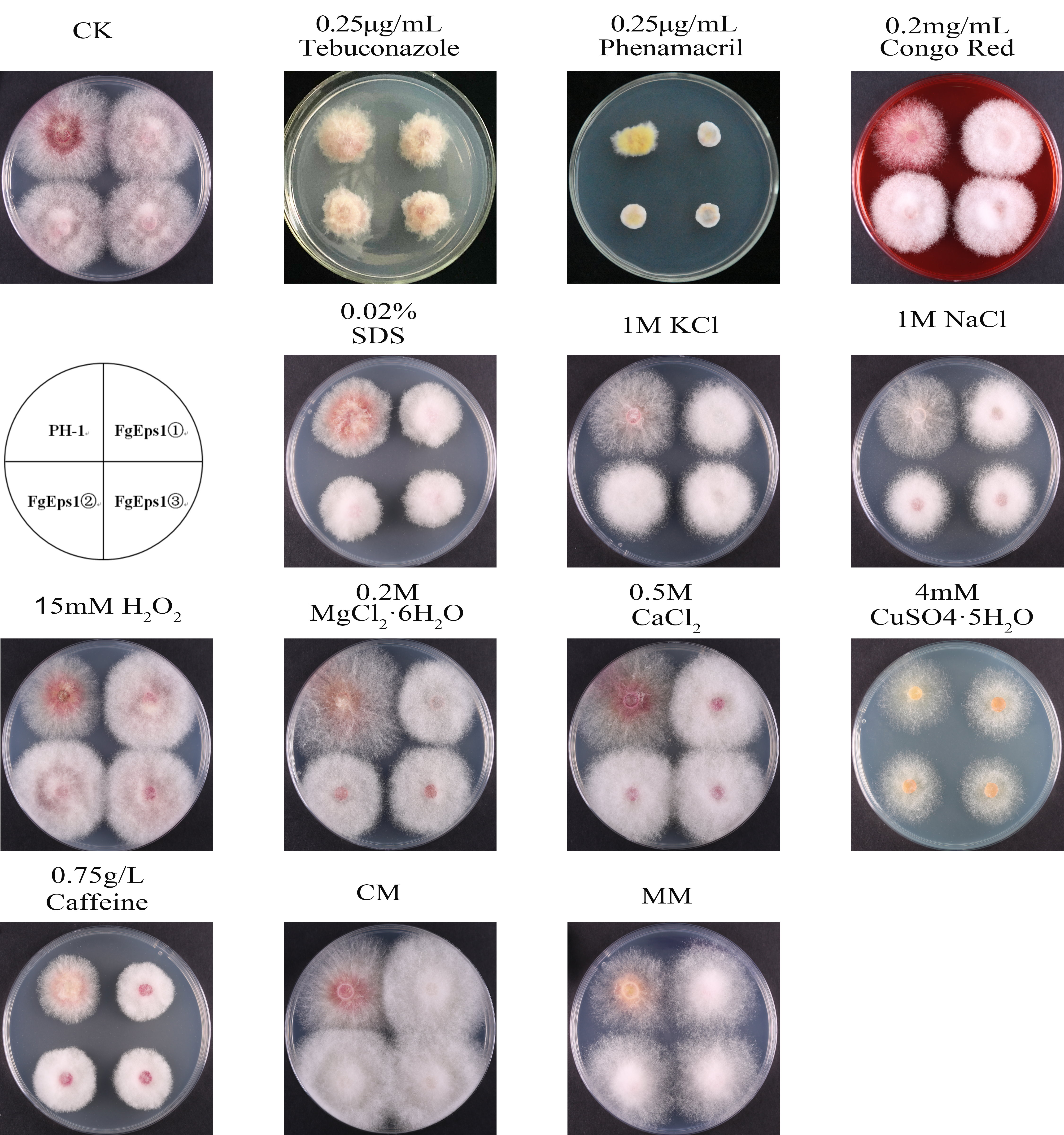

Supplement: Supplementary file 1 [file jof-09-01009-s001.zip › Figure S6.PNG]
